# Beijing/W *Mycobacterium tuberculosis* in Italy

**DOI:** 10.3201/eid1005.031024

**Published:** 2004-05

**Authors:** Nicoletta Lari, Laura Rindi, Daniela Bonanni, Enrico Tortoli, Carlo Garzelli

**Affiliations:** * Università di Pisa, Pisa, Italy; † Ospedale Careggi, Firenze, Italy

**Keywords:** Tuberculosis, Mycobacterium tuberculosis, Beijing/W strains, Molecular epidemiology, IS6110 RFLP, Spoligotyping

**To the Editor:** Molecular typing of *Mycobacterium tuberculosis* strains isolated in several countries in recent years has shown that a group of strains known as “Beijing” is widespread around the world (*1*). The Beijing group of *M. tuberculosis* has been associated with drug resistance; one multidrug-resistant strain, designated “W,” was found in New York City in the early 1990s and caused large institutional outbreaks of tuberculosis (TB) in the United States (*2*). *M. tuberculosis* strains of Beijing/W genotype are mostly prevalent in Asia (*1*), but recent data suggest that they have been spreading in Indochina and are prevalent among younger persons in Vietnam (*3*). Beijing/W strains are also widespread in Eastern Europe (*1*); during the last decade, the Beijing/W genotype of *M. tuberculosis*, with more prevalent drug-resistant mutations than non-Beijing strains, has been identified in 40% to 50% of clinical isolates studied in Russia (*4*).

We studied a total of 245 *M. tuberculosis* strains collected during a 1-year period, from January to December 2002, from the same number of TB patients hospitalized in Tuscany, Italy. All the isolates were typed by the standardized IS*6110* restriction fragment length polymorphism (RFLP) and the spoligotyping (spacer oligonucleotide typing) techniques. A total of 216 distinct IS*6110* RFLP patterns were found among the 245 isolates; 51 isolates (20.8%) occurred in 23 clusters, each constituting strains with an identical IS*6110* RFLP and spoligotype pattern; 19 clusters contained two isolates each, 3 contained three isolates, and 1 contained four isolates. Spoligotype analysis showed seven isolates with the typical Beijing/W pattern of probe hybridization only to spacer sequences 35–43. The Beijing/W isolates yielded distinct IS*6110* RFLP profiles with similarity coefficient >57.8%. Characteristics of the Beijing/W strains and respective patients, obtained from clinical records, are reported in the Table. Although the overall prevalence of Beijing/W strains was low (7/245, 2.9%), five of the seven strains were from recent immigrants to Italy from China who live in the same area; the other two strains were from Italian citizens also living in that area. Recent immigration from high-prevalence areas is therefore likely to be associated with the occurrence of the Beijing/W genotype in Italy. None of the Beijing/W strains was associated with TB outbreaks; nonetheless, infection of Italian residents with Beijing strains suggests that spread of this genotype is ongoing.

**Table:** Characteristics of *Mycobacterium tuberculosis* strains of Beijing/W genotype isolated in 2002 in Tuscany, Italy^a^

Strain no.	Patient’s country of birth	Sex	Age	HIV status	Years in Italy	Site of TB	Drug resistance^b^				
							Str	Inh	Rif	Eth	Pza
669	China	M	40	–	1	Pulmonary	S	S	S	S	S
763	China	M	42	–	<1	Pulmonary	S	S	S	S	S
804	China	F	23	–	4	Pulmonary	NT	S	S	S	S
836	China	M	34	–	1	Pulmonary	NT	S	S	S	S
884	Italy	F	39	+	NA	Extrapulmonary	NT	S	S	S	S
952	Italy	F	28	–	NA	Pulmonary	NT	R	S	S	S
974	China	F	27	–	1	Pulmonary	NT	S	S	S	S

^a^TB, tuberculosis; Str, Sterptomycin; Inh, isoniazid; Rif, rifampin; Eth, ethambutol; Pza, pirazinamide; S, susceptible; R, resistant; M, male; F, female; NA, not applicable; NT, not tested. ^b^Drug resistance was assessed by the radiometric BACTEC system (Becton Dickinson, Towson, MD) according to the proportion method.

Beijing/W strains have been strongly associated with drug resistance in a number of countries (*2*,*4*–*6*), but elsewhere the association was weak or absent. In our survey, no substantial drug resistance was observed; all Beijing/W strains isolated in Tuscany were susceptible to rifampin, ethambutol, pirazinamide, and streptomycin (tested only in two strains), and all but one were susceptible to isoniazid.

Although we detected only a few cases, our data do not show a trend of Beijing/W strains’ being associated with infection in young people, as has been observed in other settings (*3*). The age of immigrants with Beijing/W TB (mean 33.2 years, standard deviation [SD] 8.2 years) did not significantly differ from that of immigrants infected with non-Beijing/W strains (30.7 years, SD 7.4 years), a find that indicates that, at least in our setting, immigrant status, rather than *M. tuberculosis* genotype, is associated with infection in young people. The few cases of Beijing/W infections in Italian-born patients do not allow us to draw conclusions regarding nonimmigrant patients.

In conclusion, *M. tuberculosis* strains of Beijing/W genotype are becoming widespread worldwide, including in countries with a low prevalence of TB. Their association with drug resistance and infection in young people, clearly shown in certain settings, remains to be defined. Further molecular epidemiologic surveillance is needed to monitor trends in prevalence and spread of these strains.
